# Rational design 2.0: transitioning from static structural biology to computational prioritization and iterative vaccine optimization for RSV

**DOI:** 10.3389/fimmu.2026.1862216

**Published:** 2026-06-22

**Authors:** Xiulong Wei, Jing Chen, Zhaolong Li

**Affiliations:** 1 Center of Infectious Diseases and Pathogen Biology, The First Hospital of Jilin University, Changchun, China; 2 Institute of Virology and Acquired Immunodeficiency Syndrome (AIDS) Research, The First Hospital of Jilin University, Changchun, China

**Keywords:** computational vaccinology, PLMS, preF, rational design 2.0, respiratory syncytial virus

## Abstract

Respiratory syncytial virus (RSV) is a major cause of severe lower respiratory tract disease (LRTD) in infants and older adults worldwide. Although vaccines based on the fusion (F) protein have shown progress, their efficacy remains limited by antigenic instability and viral evolution. The metastable transition of the F protein between prefusion (preF) and postF conformations critically determines the exposure of neutralizing epitopes, with most potent antibodies targeting preF–specific sites. In addition, the glycosylated G protein contributes to immune evasion through glycan shielding and CX3C-mediated immunomodulation. Recent advances in structural biology and computational protein design have improved the stabilization of preF conformations; however, these approaches do not fully address antigenic variability. Emerging methods, including protein language models (PLMs) and structure prediction frameworks, enable antigen design to be guided by sequence–structure relationships, allowing researchers to prioritize candidate antigens with favorable stability profiles. Here, we propose the term “Rational Design 2.0” to describe this emerging framework. By integrating structural information with evolutionary and sequence-level constraints, Rational Design 2.0 extends RSV vaccine design beyond static structural optimization and provides a conceptual framework for future vaccine-development strategies.

## 1 Introduction

**Figure 1:**
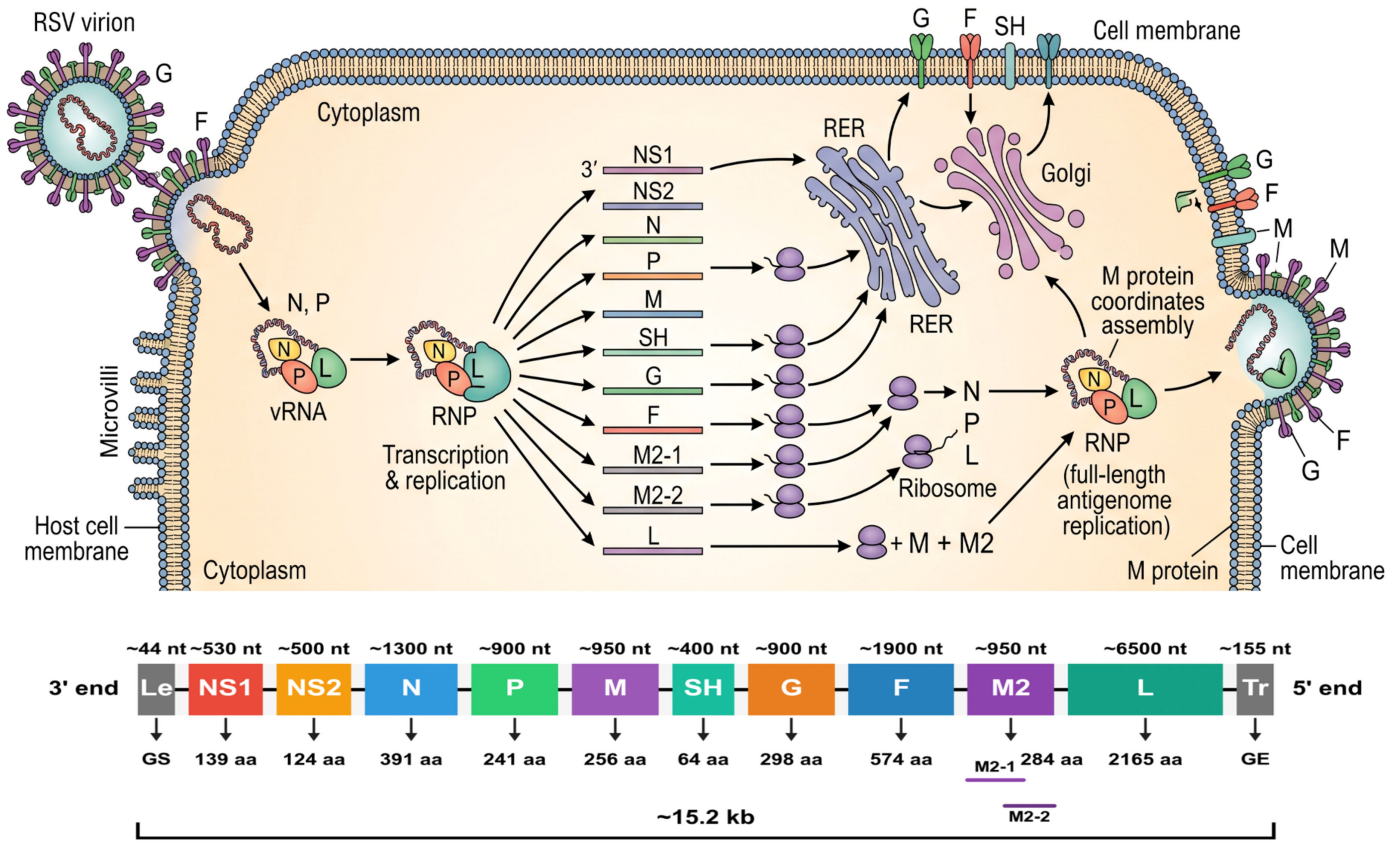
Cytoplasmic life cycle and genome organization of RSV. (Top) Viral life cycle. The RSV life cycle occurs entirely in the cytoplasm. Infection is initiated by attachment of the viral glycoprotein G to cellular receptors, followed by F-mediated fusion with the plasma membrane. The incoming viral ribonucleoprotein (vRNP) complex, composed of genomic RNA encapsidated by nucleoprotein (N) and associated with phosphoprotein (P) and the polymerase subunit L, is released into the cytoplasm to initiate transcription and genome replication. Viral glycoproteins (G, F, and SH) are processed through the secretory pathway and trafficked to the plasma membrane, whereas internal proteins (N, P, L, and M) accumulate in the cytoplasm. During late infection, the matrix protein (M) oligomerizes beneath the plasma membrane to coordinate assembly by linking vRNP complexes with surface glycoproteins, driving virion budding. (Bottom) Genome architecture. RSV contains a ~15.2 kb non-segmented, negative-sense RNA genome organized from the 3′ leader (Le) to the 5′ trailer (Tr), encoding 10 mRNAs and 11 proteins. The M2 locus produces M2–1 and M2–2 from overlapping open reading frames. Transcription follows a polymerase-driven start–stop mechanism, generating a 3′-to-5′ gradient in mRNA abundance. Genes proximal to the 3′ end, including NS1 and NS2, are expressed at high levels early in infection, enabling rapid suppression of host antiviral responses.

Respiratory syncytial virus (RSV) is a non-segmented, negative-sense single-stranded RNA virus of the family Pneumoviridae and genus Orthopneumovirus (1). Its genome encodes various structural proteins (F, G, SH, M, N, P, L) and non-structural proteins (NS1, NS2), which drive viral entry, replication, and immune regulation (**Figure 1**) (2). RSV is one of the leading pathogens causing lower respiratory tract infections in infants and young children worldwide, resulting in approximately 33 million infections, over 3 million hospitalizations, and about 60,000 child deaths annually (3); it also imposes a significant disease burden among the elderly and immunocompromised populations, with hospitalization rates approaching those of influenza, establishing its major public health significance (4).

During viral entry, the fusion protein F, as a typical Class I fusion protein, serves as the key molecule mediating the fusion of the viral envelope with the host cell membrane (5). The F protein is synthesized in the form of the precursor F0, which is cleaved by proteases to generate the F1 and F2 subunits and assemble into a trimer; it promotes viral attachment and endocytosis by cooperatively engaging host receptors (such as NCL, IGF1R, etc.) (6, 7). The F protein transitions between a metastable prefusion (preF) conformation and a stable postfusion (postF) conformation (8). The preF conformation specifically exposes highly neutralizable epitopes (such as Site Ø and Site V), whereas these key epitopes are lost during the transition to the postF conformation, resulting in a significant decrease in antigenicity (9). Therefore, the preF conformation has been established as the core immunological target for inducing potent neutralizing antibodies.

In contrast, the G protein primarily mediates viral attachment and exhibits high variability; its CX3C motif binds to CX3CR1 and can interfere with the immune response through its soluble form (10). However, due to significant antigenic drift and the risk of immunopathology, it is not suitable as a core vaccine antigen (11, 12). The NS1 and NS2 proteins achieve potent immune evasion by inhibiting the interferon pathway and are key virulence factors for the virus to establish early infection (13, 14). The SH protein, acting as a virion-associated pore protein, participates in ion homeostasis regulation and anti-apoptotic processes; its deletion enables viral attenuation while preserving immunogenicity (15–18). The P and L proteins form the core of the viral replication complex; they promote efficient replication and evade immune recognition by forming inclusions through liquid-liquid phase separation (19–21), while the M protein governs viral assembly and budding and participates in the suppression of host transcription (22).

Early vaccine efforts illustrate these constraints. Formaldehyde-inactivated RSV failed to preserve the preF conformation and induced predominantly non-neutralizing antibodies, resulting in vaccine-associated enhanced respiratory disease (VAERD) (9, 23). Structure-based antigen design has since improved immunogenicity by stabilizing the preF state and enabled multiple vaccine candidates to enter clinical development (24). However, approaches based on a single stabilized conformation remain limited in their ability to address antigenic drift (25). Recent advances in protein language models and structure prediction are beginning to enable antigen design at the sequence level within integrated sequence–structure frameworks (26, 27). These approaches allow for candidate prioritization based on stability and immune escape, and can be combined with delivery platforms such as recombinant proteins, mRNA, and nanoparticles to further shape immune responses (28, 29). Here, we review recent advances in RSV vaccine design, focusing on how antigen structure, immune recognition, and viral evolution intersect, and discuss how computational approaches may facilitate stronger immune responses and broadly protective vaccines.

## 2 Fundamental viral structure of RSV

### 2.1 F protein

The F protein is a class I fusion protein that mediates fusion between the viral envelope and the host cell membrane (30). It is synthesized as an inactive precursor (F0) and cleaved by furin-like proteases to release the p27 peptide, generating the disulfide-linked F1 and F2 subunits that assemble into a functional homotrimer (31). The F protein interacts with multiple host factors, including nucleolin (NCL), epidermal growth factor receptor, insulin-like growth factor-1 receptor (IGF1R), and intercellular adhesion molecule-1, which facilitate viral attachment, endocytosis, and membrane fusion (7, 32–35). Engagement of IGF1R activates downstream signaling pathways, including PKCζ, promoting recruitment of NCL to the plasma membrane, where it functions as a cofactor for viral entry (7, 36). At the cell surface, NCL binds the F protein to stabilize viral attachment (37), while IGF1R signaling also induces actin remodeling to promote endocytosis and triggers conformational rearrangement of the F protein, exposing the fusion peptide and enabling membrane insertion and fusion (7, 38–40). During later stages of infection, RSV downregulates IGF1R expression to limit superinfection (7). Although F gene expression occurs in the cytoplasm, it utilizes cryptic polyadenylation and splicing signals, reflecting an additional layer of post-transcriptional regulation (41–43).

The F protein is relatively conserved between RSV A and B subtypes and represents the primary target of neutralizing antibodies, making it a central antigen in vaccine design (44–46). Structurally, it transitions between a metastable preF conformation and a stable PostF state (39). The PreF conformation exposes multiple highly neutralizing epitopes, including sites Ø and V, which are conformation-specific and dominate the neutralizing activity of human sera (9, 47). In contrast, shared epitopes (sites I–IV) are present in both conformations but contribute less to potent neutralization. Following receptor engagement, the F protein undergoes irreversible conformational rearrangement into the PostF state, accompanied by reduced accessibility of several prefusion-specific neutralizing epitopes and altered antigenic properties (48–51). This transition is a structural requirement for viral membrane fusion and entry rather than an active immune-evasion mechanism. The reduced recognition of key neutralizing epitopes represents a downstream consequence of the conformational rearrangement required for membrane fusion. Consistent with this, *in vitro* studies show that stabilized PreF antigens capture more than 90% of serum neutralizing activity, whereas PostF antigens bind only ~30% (9, 52). Epitope mapping further indicates that site Ø alone can account for up to 35% of total neutralizing activity, with site V also serving as a major target for high-affinity cross-neutralizing antibodies (9, 52–54).

Such biophysical constraints have important implications for vaccine efficacy; accordingly, immunogens predominantly presenting postF or unstable conformations generally exhibit reduced protective immunogenicity. For example, the recombinant RSV F nanoparticle vaccine (ResVax) developed by Novavax primarily presents postF or mixed conformations with limited exposure of site Ø (50, 55). In a global Phase III trial involving 4,636 pregnant women, this vaccine failed to meet its primary endpoint, achieving only 39.4% efficacy against RSV-associated lower respiratory tract infection in infants (55). Consequently, stabilization of the preF conformation and preservation of vulnerable epitopes, including sites Ø and V, remain central determinants of protective immunity.

### 2.2 The attachment (G) protein

The RSV G protein is a type II transmembrane glycoprotein characterized by extensive glycosylation and high sequence variability, which contribute to its roles in host cell attachment and immune evasion (56, 57). It contains a conserved central core region (Gcc) of ~40 amino acids flanked by heavily glycosylated mucin-like domains (58, 59). During early stages of infection, the membrane-anchored G protein facilitates viral attachment by interacting with host cell surface glycosaminoglycans through its heparin-binding domain (60). In addition, the disulfide-stabilized CX3C motif within the Gcc specifically engages the CX3CR1 receptor on airway epithelial cells, promoting efficient viral binding (61, 62). In contrast to the relatively conserved F protein, the G protein exhibits genetic variability across RSV strains (63). Recurrent insertion events, including the 24–amino acid duplication in subtype A and the 20–amino acid repeat in subtype B, alter its structural conformation and antigenic accessibility, contributing to immune evasion (63, 64). RSV further modulates host immunity through the production of a soluble G protein (sG), generated by alternative translation initiation at methionine 48 (65, 66). This secreted form retains the CX3C motif and mimics the host chemokine CX3CL1. By binding CX3CR1 on leukocytes, sG interferes with endogenous CX3CL1 signaling, reducing immune cell recruitment and dampening interferon responses (60, 67, 68). In addition, sG can function as an antibody decoy by binding G-specific antibodies, limiting their access to virion-associated antigens (57, 69, 70).

Experimental studies have highlighted the immunopathological risks associated with the G protein. Vaccination with native G protein containing intact mucin-like domains induces a strongly Th2-biased response in animal models, which is associated with enhanced disease upon RSV challenge (71). Analyses of bronchoalveolar lavage fluid (BALF) show marked increases in eosinophil infiltration, rising from baseline levels (<3%) to 14–25% following infection (72, 73). Despite its importance in viral attachment and immune modulation, the G protein elicits relatively low neutralizing antibody responses and is subject to antigenic drift. Its potential to interfere with host immunity and to promote immunopathology further limits its suitability as a primary vaccine antigen. Accordingly, current vaccine strategies focus on the stabilized preF protein as the principal target for inducing protective immunity.

### 2.3 “Beyond F and G: alternative viral targets for RSV vaccine design”

Beyond the surface glycoproteins F and G, several internal and non-structural RSV proteins contribute to immune evasion, viral replication, and pathogenicity, informing rational strategies for vaccine attenuation and antiviral development. Among these, the non-structural proteins NS1 and NS2 function as major antagonists of type I and III interferon (IFN) signaling, suppressing host antiviral responses at multiple levels to facilitate early viral replication (74–81). Their central role in virulence has made NS gene deletion a widely explored strategy in live-attenuated vaccine (LAV) development, enabling viral attenuation while preserving immunogenicity (82–86).

At the core of the replication machinery, the P and the L protein coordinate viral RNA synthesis and transcription (87–92). Temperature-sensitive mutations in the L gene have been incorporated into multiple LAV candidates to restrict viral replication and improve safety profiles (93–95). Similarly, the SH protein contributes to immune modulation and cellular homeostasis, and deletion of SH has been explored as an attenuation strategy in vaccine design (15–17, 96). The M protein additionally contributes to viral assembly and budding through coordination of viral ribonucleoprotein complexes and envelope glycoproteins (97–104).

Although these proteins are not dominant targets of neutralizing antibodies, mechanistic insights into their biological functions have informed rational attenuation strategies that complement antibody-focused vaccine approaches. Nevertheless, the limited efficacy of vaccine strategies relying predominantly on cellular immunity highlights the complexity of RSV vaccine development. For example, the Phase III failure of the T cell–based vaccine VANIR, targeting the highly conserved M protein, suggests that cellular immune responses alone may be insufficient for strong protection in the lower respiratory tract (105–107). Effective RSV vaccine engineering therefore requires balancing viral attenuation with the induction of both cellular immunity and potent neutralizing antibody responses.

## 3 The evolutionary trajectory of RSV vaccines

### 3.1 The empirical era: conformational collapse and the immunopathology of VAERD

Early efforts to develop an RSV vaccine were unsuccessful, largely due to a limited understanding of the conformational properties of the F protein, particularly the instability of its preF state (108). The formaldehyde-inactivated RSV (FI-RSV) vaccine developed in the 1960s followed a traditional empirical approach, relying on chemical inactivation without preserving antigenic structure (109). From a structural immunology perspective, this failure reflects an inability to maintain the conformational integrity of the target antigen (110). Formaldehyde treatment disrupts the metastable preF protein, promoting its irreversible transition to the more stable postF conformation (111).

This conformational change alters the antigenic landscape of the viral surface. The immune response elicited by FI-RSV is therefore directed primarily against epitopes present on the postF conformation, which have limited neutralizing capacity. Key preF–specific neutralizing epitopes, including site Ø, are lost during this transition (39). As a result, vaccinated individuals fail to generate sufficient high-affinity neutralizing antibodies. Upon subsequent infection, viral replication is not effectively controlled, while non-neutralizing antibodies form immune complexes that contribute to immunopathology. This response is characterized by Th2-biased cytokine production, complement activation, and pulmonary eosinophil infiltration (112, 113), a syndrome known as VAERD.

The failure of FI-RSV highlights a central limitation of traditional vaccine approaches when applied to metastable viral proteins (114). It also establishes a key principle for RSV vaccine design: protective efficacy and safety depend on maintaining the correct antigen conformation and preserving neutralization-sensitive epitopes.

### 3.2 Rational design 1.0

To address the instability of antigen conformation in traditional vaccine design, structure-based approaches have introduced physical constraints to stabilize specific protein states. In 2013, the preF trimer was resolved at atomic resolution by combining cryo-electron microscopy and X-ray crystallography, defining the structural basis of key neutralizing epitopes, including site Ø (39). Based on these structural insights, engineered disulfide bonds and cavity-filling mutations were introduced to stabilize the preF conformation (DS-Cav1 design), increasing the energetic barrier to conformational rearrangement and favoring retention of the antigenically relevant state (24). This strategy enabled the development of vaccines that present a defined and immunogenic conformation.

**Figure 2:**
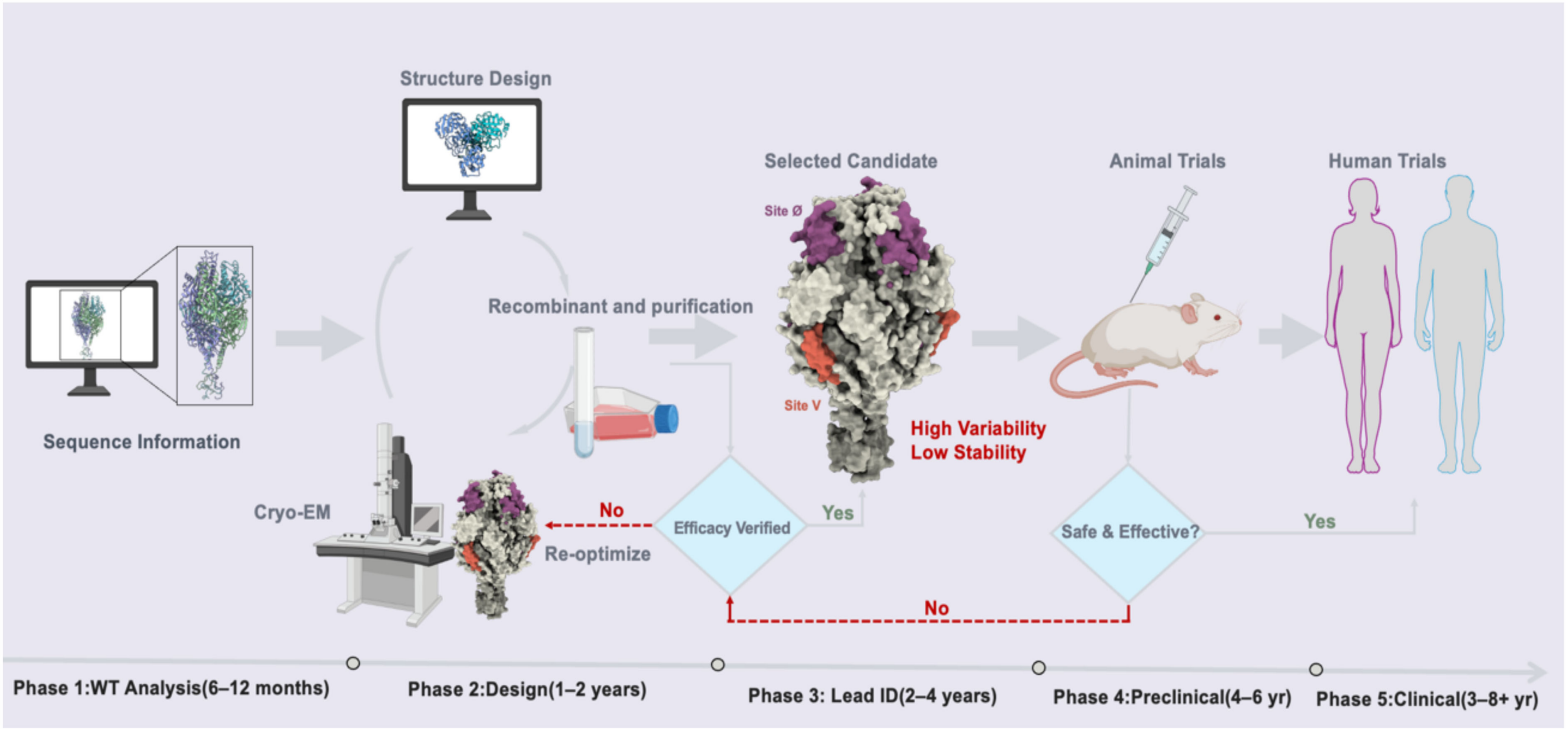
The rational design 1.0 paradigm: structure-based stabilization. Atomic-resolution structures of the RSV preF glycoprotein enabled the rational stabilization of key neutralizing epitopes, including site Ø. Designs such as DS-Cav1 increased the energetic barrier to refolding and preserved the antigenically relevant preF conformation, forming the basis of current RSV vaccines, including Arexvy, Abrysvo, and mRNA-1345. However, stabilization of a single conformational state remains vulnerable to antigenic drift. Sequence variation at site V and distal substitutions such as L305I can alter epitope accessibility through long-range conformational effects, influencing antibody recognition at multiple neutralization-sensitive sites. These limitations highlight the need for vaccine design frameworks that integrate structural, evolutionary, and sequence-based information to better anticipate antigenic change.

These advances translated rapidly into clinical success. Recombinant protein vaccines such as Arexvy and Abrysvo, as well as mRNA-based vaccines including mRNA-1345, all incorporate stabilized preF antigens and have demonstrated strong neutralizing antibody responses and clinical protection (**Figure 2**) (115–117). These results show that stabilizing antigen structure is sufficient to overcome the intrinsic instability of metastable viral proteins (118). Despite these successes, conformation-stabilizing strategies have inherent limitations. By fixing the antigen in a single structural state, they remain vulnerable to antigenic drift (119). Even minor mutations, such as changes at site V in RSV subtype B, can alter epitope structure and reduce antibody binding (119). Antigenic variation may arise not only from mutations located directly within antibody-binding interfaces but also from distal substitutions that alter epitope accessibility through conformational coupling. For example, a recent study demonstrated that the L305I substitution can modulate multiple neutralization-sensitive epitopes, including sites Ø, II, and IV, through long-range structural effects (120). These findings highlight the limitations of relying solely on static structural interpretation and support the use of sequence- and structure-informed computational approaches to prioritize variants with potentially altered antigenic properties for downstream experimental validation. The loss of efficacy of the monoclonal antibody suptavumab illustrates the impact of such variation (121). In addition, a single stabilized conformation may not adequately capture sequence diversity or sustain long-term protection as immune responses wane (115, 121).

Structural stabilization alone does not fully address the antigenic diversification observed among contemporary RSV strains, motivating vaccine-design strategies that additionally incorporate sequence-level variation and evolutionary dynamics, moving beyond static structural optimization toward more hypothesis-guided frameworks (122).

### 3.3 Rational design 2.0: the computational prioritization and frontier

Rational Design 2.0 extends structure-based vaccine design by integrating evolutionary information derived from protein language models (PLMs) with structural prediction frameworks. This framework supports candidate selection before experimental evaluation, shifting vaccine design from retrospective structural interpretation toward computational exploration of sequence space.

**Figure 3:**
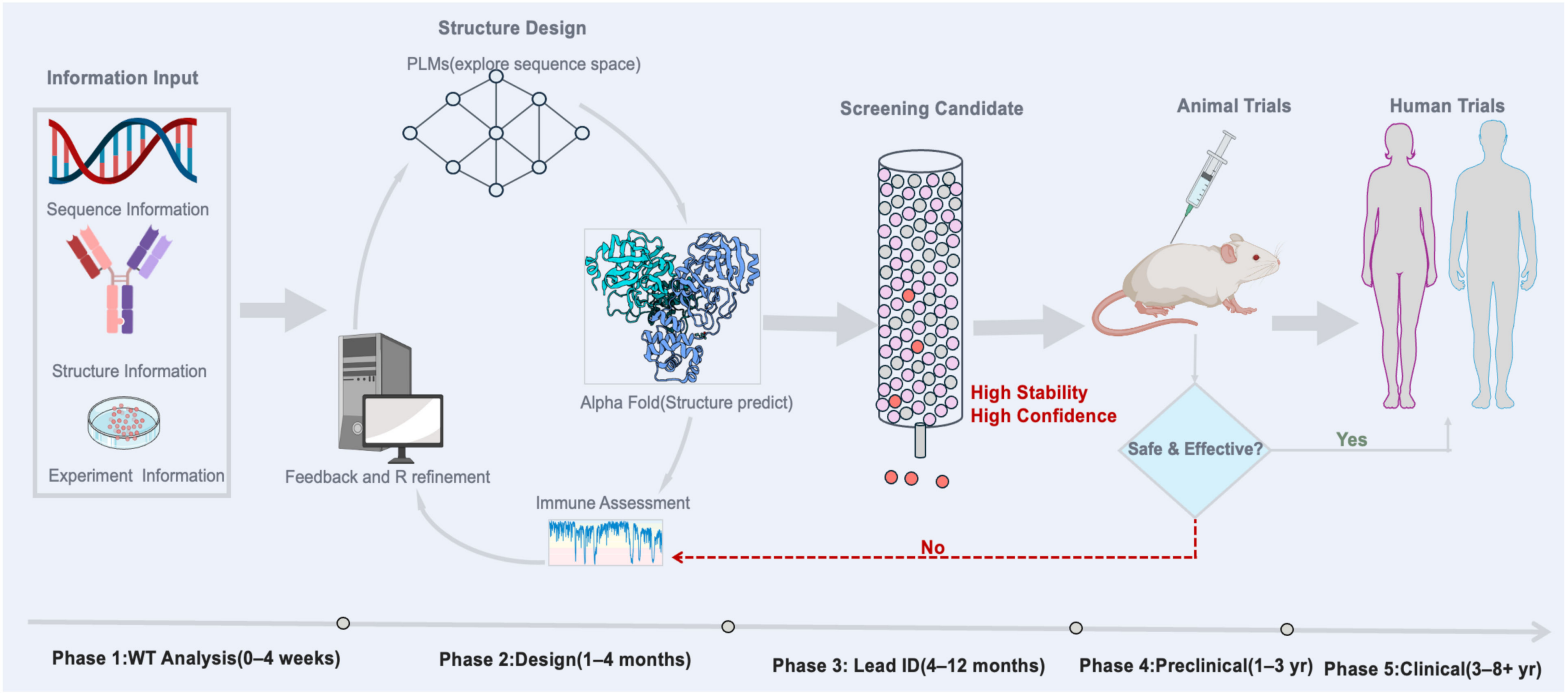
The rational design 2.0 paradigm: AI-driven computational vaccinology. Sequence, structural, and experimental information are integrated to train PLMs that explore antigen sequence space beyond naturally occurring variants. Candidate sequences are evaluated through structure prediction and immunological screening, while experimental measurements are iteratively fed back into the design process to refine model performance and guide subsequent rounds of optimization. This closed-loop framework enables the identification of antigen candidates with enhanced stability, manufacturability, and immunogenic potential before progression to preclinical and clinical evaluation. Unlike traditional approaches that focus on stabilizing a single antigenic conformation, AI-enabled design leverages sequence–structure relationships and experimental feedback to anticipate antigenic variation and accelerate the discovery of broadly protective vaccine antigens.

Compared to Rational Design 1.0, which relies predominantly on experimentally resolved structures for rationalization, Rational Design 2.0 operates as a computational pre-screening framework for exploring sequence diversity and identifying prioritized candidate antigens. Nevertheless, current computational frameworks remain subject to important biological and structural limitations. Most sequence-based PLMs primarily infer evolutionary constraints from amino acid sequences and therefore incompletely capture higher-order properties such as glycan dynamics, steric shielding, conformational heterogeneity, and the structural complexity of heavily glycosylated mucin-like domains. These limitations are particularly relevant for RSV glycoproteins, in which dense glycan shielding and metastable conformational transitions influence epitope accessibility and immune recognition (123). In addition, many current structural prediction frameworks still rely on relatively static conformational representations and may inadequately model transient or ensemble-dependent antigenic states. As a result, traditional “snapshot-based” analyses are being complemented by models that capture conformational variability and dynamic protein behavior. Recent work suggests that models such as AlphaFold2 can partially represent conformational diversity by leveraging evolutionary information, extending structure prediction beyond single static states (**Figure 3**) (124). These approaches increasingly allow viral antigens to be analyzed in the context of their molecular interactions with antibodies and host factors (125).

Sequence-based models further provide insight into evolutionary constraints. Protein language models can infer fitness landscapes directly from sequence data, where mutations that preserve sequence likelihood tend to maintain function, whereas deviations may indicate antigenic change. This framework can identify mutations associated with both viral fitness and immune recognition and has shown utility in modeling mutation-associated changes in systems such as the SARS-CoV-2 spike protein (AUC ≈ 0.85) (27). Extensions to protein complexes allow evaluation of mutation effects at interaction interfaces. Models such as PLM-interact capture inter-chain residue relationships and improve assessment of binding changes across species (with AUPR improvements of up to 28%) (126). Similarly, MINT enables quantitative estimation of mutation-induced changes in binding free energy (ΔΔG), improving performance on the SKEMPI benchmark by ~29% (127). In viral systems, these predictions show partial correlation with experimentally measured neutralization datasets, including IC_50_ measurements, with ~80% accuracy in identifying persistent neutralizing responses under defined thresholds. These approaches support the prioritization of variants according to predicted changes in molecular interactions and antibody recognition, thereby generating testable hypotheses for subsequent experimental assessment of immune escape.

Although experimental validation remains indispensable, computational workflows can reduce the number of candidates entering downstream testing (128). By prospectively filtering thermodynamically unstable or potentially immune-evasive immunogens during the in silico design phase, AI-assisted pipelines can shift experimental efforts from broad empirical screening toward the targeted evaluation of more refined candidates (129). Whether these approaches ultimately reduce late-stage attrition remains to be established through prospective studies. Beyond sequence-level prioritization, computational vaccinology may also facilitate the identification of structural features associated with protective immunity. Through the integration of PLMs, structural prediction frameworks, and epitope analysis, these approaches can assist in identifying antigen configurations that favor conformational stability and preserve the accessibility of key neutralizing epitopes (130, 131). For pathogens such as RSV, in which protective immunity is highly dependent on conformation-sensitive antigenic sites, structure-guided and sequence-constrained design strategies may improve the retention of vulnerable neutralizing epitopes and support the development of more immunogenically favorable candidates (110). Whether these improvements translate into enhanced protective immunity remains to be experimentally determined, particularly by prioritizing immunogens with improved structural and antigenic properties. Metastable structural parameters ultimately remain tools for candidate prioritization and hypothesis generation rather than standalone predictors of vaccine efficacy. Their contribution to protective immunity remains dependent on systematic *in vitro*, *in vivo*, and clinical validation (128, 129).

Overall, RSV vaccine development is transitioning from empirically guided approaches toward computationally assisted frameworks that integrate antigen structure, evolution, and immune recognition. Computational models do not replace experimental validation but instead provide a means of generating and refining testable hypotheses prior to empirical testing.

## 4 RSV vaccine modalities and clinical pipelines

As a primary pathogen of severe lower respiratory tract infections in infants and older adults worldwide, the framework for RSV vaccine development is undergoing a critical transition from overcoming antigenic conformational instability to addressing viral evolutionary diversity. By integrating protein language models with structure prediction frameworks, the research trajectory is shifting from static structural optimization toward the “Rational Design 2.0” era. To better understand this trajectory, the diverse vaccine modalities discussed below can be mapped to the three developmental eras. The conventional inactivated vaccines represent the Empirical Era, characterized by structural collapse. Recombinant proteins, live-attenuated vaccines (LAVs), and viral vectors primarily embody Rational Design 1.0, relying on static physical stabilization of the preF conformation. In contrast, emerging nanoparticle and computationally optimized mRNA platforms mark the transition toward Rational Design 2.0, where sequence-structure-immunogenicity relationships are prospectively engineered, providing a systematic framework for prioritizing.

### 4.1 Recombinant protein and subunit vaccines

Representing the pinnacle of Rational Design 1.0, these vaccines primarily rely on mammalian cell lines for expression and purification. They target the F protein, introducing mutations such as DS-Cav1 to physically anchor the antigen in the highly immunogenic PreF metastable state, thereby maximizing exposure of Site Ø to induce highly effective neutralizing antibodies (39). Representative products, Arexvy (GSK) and Abrysvo (Pfizer) (**Table 1**), have both been approved, with protective efficacy against severe lower respiratory tract disease in the elderly exceeding 82% (115, 116).

**Table 1:** The evolving landscape of respiratory syncytial vaccines and therapeutics.

Platform	Product	Molecular target	Mechanism of action	Efficacy/status
Subunit	Arexvy (GSK)	PreF (Site Ø/V)	DS-Cav1 stabilized; Prevents fusion	> 82.6% (Approved)
	Abrysvo (Pfizer)	PreF (Site ø)	Bivalent (A/B); Maternal immunization	88.9% (Approved)
mRNA	mRESVIA (Moderna)	PreF (Site 0)	LNP-encapsulated mRNA; In-situ translation	83.7% (Approved)
LAV	rA2ΔSH	NLRP3/Apoptosis	SH deletion; Pro-inflammatory shift	Phase I/II
	6120/ΔNS2	IFN-α/ß Pathway	NS2 deletion; Relieves IFN antagonism	Phase I/II
Vector	MVA-BN-RSV	F/N/M/M2-1	Viral vector; Robust CD8+ T-cell priming	42.9% (Ph III)
Nanoparticle	IVX-121	PreF (Trimeric)	Icosavax VLP; BCR cross-linking	~10x Neut. titers
MAb	Nirsevimab	PreF (Site ø)	Long-acting (YTE mod); Passive immunity	79.5% (Approved)
	3D3	G (CCD/CX3C)	sG-decoy neutralization; CX3CR1 blocking	Pre-clinical

Summary of contemporary RSV intervention platforms, molecular targets, and clinical efficacy. BCR, B-cell receptor; DS-Cav1, disulfide-cavity 1 stabilizing mutations; IFN, interferon; LAV, live-attenuated vaccine; LNP, lipid nanoparticle; MAb, monoclonal antibody; NLRP3, NLR family pyrin domain containing 3; PreF, pre-fusion conformation of the F protein; RdRp, RNA-dependent RNA polymerase; sG, soluble G protein; VLP, virus-like particle; YTE, M252Y/S254T/T256E Fc-region mutation.

### 4.2 mRNA-based vaccines

This platform does not inject whole proteins; instead, it encapsulates codon-optimized preF mRNA within LNPs. The LNPs are delivered into cells to enable *in situ* translation of the antigen (132), naturally activating innate immune pathways and inducing a Th1-biased cellular and humoral immune response (133). The representative product, mRESVIA, was approved in 2024 and demonstrated an efficacy of 83.7% in protecting the elderly (**Table 1**) (117).

### 4.3 Live-attenuated vaccines

Designed specifically for seronegative infants and young children lacking immune imprinting. Their preparation relies on reverse genetics to perform “precision genetic surgery” (134): targeting the knockout of immune evasion factors to lift the local mucosal interferon blockade (e.g., NS1/NS2) (83); or knocking out the replication switch (M2–2 protein) to lock the virus in a state of high antigen expression (135). Several candidate strains (e.g., rA2ΔSH, RSV/6120/ΔNS2) have demonstrated good infectivity and immunogenicity in Phase I/II clinical trials (**Table 1**), inducing neutralizing antibodies and memory immune responses. Because they are administered via the intranasal route and replicate locally in the respiratory tract, these live-attenuated vaccines are considered to have the potential to activate respiratory mucosal immunity (83, 136).

### 4.4 Viral vector vaccines

Highly conserved internal proteins (N, M2-1, M) and the F protein were encoded into adenoviral or modified vaccinia virus vectors (107); through intracellular expression of the antigens and presentation via the MHC class I pathway, the aim was to induce potent CD8^+^ T cell-mediated cellular immunity (137). However, the representative candidate vaccine MVA-BN-RSV demonstrated only 42.9% efficacy against RSV-associated lower respiratory tract disease in Phase III clinical trials, failing to meet the primary endpoint; this insufficient protection was attributed to limited levels of neutralizing antibodies (**Table 1**) (105). Although viral vector vaccines can induce cellular immune responses, their protective effect against RSV-associated lower respiratory tract disease remains limited in the absence of synergistic high-level neutralizing antibodies.

### 4.5 Nanoparticle and peptide-based vaccines

As a bridge to Rational Design 2.0, nanoparticle vaccines rely on a computational structural design platform. For instance, IVX-121 utilizes the spatial structure of a protein scaffold to geometrically amplify the B-cell cross-linking effect of PreF, resulting in neutralizing antibody titers approximately 10-fold higher than those of the monomer (**Table 1**) (28); DPX-RSV, on the other hand, targets the extracellular domain of the SH protein to induce antigen-dependent cell-mediated phagocytosis (ADCP), representing a breakthrough in high-density antigen presentation (**Table 1**) (138, 139).

### 4.6 Conventional inactivated vaccines: the empirical era

Historically, FI-RSV irreversibly disrupted the F protein structure due to chemical reagents (causing it to collapse into the PostF conformation) (**Table 1**) (111), resulting in the production of large amounts of non-neutralizing antibodies after vaccination, which triggered lethal immunopathological damage (VAERD) during secondary natural infection (113). Although this platform has been phased out, it established the design principles of “conformation locking” and avoiding Th2 polarization for modern vaccines (113).

## 5 Safety assessment and computational paradigms in vaccine design

### 5.1 Immunological safety and computational risk mitigation

Safety remains a central consideration in vaccine and immunotherapeutic design (1). A major source of risk arises from immune polarization imbalance, which can lead to hypersensitivity, cytokine storm, or VAERD (113, 140). These outcomes are often linked to Th2-biased responses or dysregulated innate immune activation, underscoring the need to control early immune signaling (141, 142). Computational approaches are increasingly used for early safety assessment (143). Structure-based approaches, including molecular docking and molecular dynamics simulations, may provide preliminary mechanistic insight into potential interactions between antigens and pattern recognition receptors such as Toll-like receptors (TLRs). However, these methods should primarily be viewed as tools for hypothesis generation and candidate prioritization rather than predictors of downstream immunological outcomes (144–146). Because immune polarization arises from complex interactions involving innate signaling, antigen presentation, and host context, definitive conclusions regarding Th1/Th2 balance, cytokine responses, or vaccine safety remain dependent on experimental validation (147, 148).

AI-assisted sequence optimization can be used to identify candidate antigens for subsequent experimental evaluation (149). Deep learning frameworks integrating sequence–structure relationships have been explored to prioritize RSV F variants for subsequent experimental testing, including evaluation of interferon and cytokine responses (150, 151). For example, certain AI-assisted mRNA vaccine candidates have demonstrated Th1-skewed immune profiles in animal models (152), characterized by IFN-γ production and CD8^+^ T cell activation, while limiting Th2-associated cytokines and reducing the risk of VAERD (153–156). However, these observations reflect experimentally measured outcomes rather than direct predictions generated by computational models.

### 5.2 Age-dependent immunological considerations

Vaccine responses vary across the human lifespan, with infants and older adults representing high-risk populations with distinct immune profiles (157). In infants, immune immaturity affects both innate and adaptive responses. Reduced antigen presentation and limited responsiveness to Toll-like receptor signaling result in insufficient production of antiviral cytokines such as IL-12 and IFN-γ, favoring Th2-biased responses (158–160). This increases susceptibility to RSV infection and the risk of immunopathology following inappropriate antigen exposure. In contrast, older adults exhibit immunosenescence combined with chronic low-grade inflammation. This is characterized by reduced naïve T-cell pools, accumulation of exhausted T cells (161), and impaired innate immune function alongside elevated basal inflammation (162). These changes limit responses to new antigens, reducing both antibody and cellular immunity and compromising vaccine durability (163).

### 5.3 The “predict–generate–verify–iterate” framework

Advances in computational immunology are shifting RSV vaccine development toward a “predict–generate–verify–iterate” framework (152, 164). This approach integrates computational modeling with experimental validation to enable continuous optimization of antigen design (165). Deep learning models can rank RSV F variants according to predicted structural and functional properties across large sequence spaces (150, 152, 166–168). Candidate antigens identified in silico are then tested experimentally in expression systems and animal models to assess immunogenicity, efficacy, and safety.

Experimental data are subsequently used to refine model parameters. In this framework, computational models are viewed as hypothesis-generation tools that guide experimental design, supporting iterative model refinement and candidate evaluation (169, 170). Active learning strategies further enhance this process by continuously updating sequence–function relationships. For example, in RSV mRNA vaccine development, multi-round experimental data have been used to improve model performance and guide antigen optimization (171–173). Within this multi-round paradigm, computational models primarily function to reduce the screening burden and prioritize candidate leads for experimental evaluation, whereas definitive validation of vaccine safety, immunogenicity, and protective efficacy ultimately relies on empirical evaluation.

### 5.4 Shared principles and virus-specific constraints in class i fusion protein design

The computational framework developed for RSV F protein design extends beyond a single pathogen and provides a generalizable paradigm for Class I viral fusion proteins (174). These proteins undergo irreversible transitions from metastable preF to stable postF conformations (175), a process that often obscures key neutralizing epitopes in the postF state (176, 177). Stabilizing the preF conformation therefore remains a central strategy in vaccine design.

While this principle is broadly shared across viral families, important biological differences remain among distinct Class I fusion proteins (178). Influenza hemagglutinin (HA) and coronavirus spike proteins both rely on related conformational transitions to mediate membrane fusion, making prefusion stabilization and epitope optimization common objectives in vaccine development (179). However, extending computational design principles across Class I fusion proteins requires careful consideration of virus-specific structural and immunological constraints. Although prefusion stabilization represents a broadly conserved strategy, influenza HA and SARS-CoV-2 spike proteins differ from RSV F in glycan shield organization, conformational dynamics, and population-level immune exposure histories (180). These differences can profoundly influence epitope accessibility, antigen stability, and vaccine responsiveness, highlighting the need for increasingly context-specific computational design frameworks (178). In coronaviruses such as SARS-CoV-2, structure-guided design has enabled stabilization of the spike protein through mutations including the 2P and HexaPro substitutions, as well as disulfide engineering and cavity-filling strategies (181, 182). These modifications improve structural stability and expression while preserving neutralizing epitopes, particularly within the receptor-binding domain (RBD) (182–184). For influenza viruses, where antigenic drift is pronounced, computational approaches have enabled a shift in focus from the variable HA head to the more conserved stem region (185, 186). Structure-guided design and scaffold-based strategies have produced HA stem immunogens capable of eliciting broad, cross-subtype protection in animal models while maintaining high stability (187, 188).

Current PLMs still rely predominantly on primary amino acid sequences, limiting their ability to accurately incorporate complex post-translational modifications such as glycosylation (189). This limitation is particularly relevant for highly glycosylated viral glycoproteins, including SARS-CoV-2 spike and Influenza HA, where dynamic glycan shielding can reshape epitope accessibility and antibody recognition (190, 191). Although next-generation platforms such as AlphaFold3 have begun integrating non-protein components into structural predictions, current models remain largely restricted to static conformational snapshots and do not fully capture the dynamic steric effects imposed by flexible glycan networks. Integrating molecular dynamics simulations with glycan-aware structural modeling therefore represents an important future direction for improving computational assessment of epitope exposure and immunogenicity (192). Current computational strategies primarily focus on molecular-level binding thermodynamics or viral fitness assessments, falling short in effectively inferring interactions with the complex host immune system. Existing algorithms still struggle to simulate the dynamic shifts of the human immune repertoire. In the context of clinical translation, inherent species differences between animal models and human populations—particularly regarding antigen presentation mechanisms, B-cell receptor (BCR) diversity, and somatic hypermutation (SHM)—exert a decisive influence on the ultimate protective efficacy of vaccines (49, 113). Together, these observations suggest a common design principle across Class I fusion proteins: effective vaccine design requires coordinated control of conformational stability and epitope presentation. Computational and AI-based approaches provide a scalable framework to achieve this goal across diverse viral systems.

## 6 Limitations and bottlenecks in computational vaccinology

Although PLMs and structural prediction frameworks have improved the early-stage prioritization of vaccine antigens, computational vaccinology continues to face important limitations in describing the dynamic and multi-scale nature of biological systems. One major challenge lies in the limited representation of post-translational modifications (PTMs) and their spatial organization. Most current deep learning architectures are trained primarily on amino acid sequences and therefore incompletely capture the highly flexible and densely glycosylated environments surrounding viral surface proteins (193). For example, the respiratory syncytial virus (RSV) attachment (G) glycoprotein contains heavily glycosylated mucin-like domains together with dense arrays of both O- and N-linked glycans. These features form a dynamic glycan shield that can influence the accessibility of key neutralizing epitopes (191). As a result, prediction frameworks relying primarily on linear sequences or static structures may not fully reflect epitope exposure under physiological conditions.

Structural dynamics represent another major limitation of current computational approaches. Although next-generation tools such as AlphaFold 3 have begun incorporating certain non-protein components, their outputs remain largely centered on relatively stable, low-energy conformations and provide limited insight into the broader conformational ensembles that exist under physiological conditions (194, 195). This limitation is particularly relevant for enveloped viruses, in which many protective epitopes are associated with transient structural states. A representative example is the RSV F glycoprotein, which undergoes a transition from a metastable preF conformation to a stable postF state during membrane fusion (24). This process shapes both viral entry and patterns of neutralizing antibody recognition. Static structural predictions therefore remain insufficient to fully capture conformational plasticity and its immunological consequences.

Beyond structural and biophysical constraints, current computational strategies also remain limited in extrapolating molecular-level properties to system-level immune outcomes (196). Existing approaches are effective at evaluating local stability changes, binding energetics (such as ΔΔG), and viral fitness landscapes, yet remain insufficient for predicting immune responses within complex host environments. In particular, processes such as B-cell receptor (BCR) repertoire diversification, somatic hypermutation (SHM) (197), and the influence of pre-existing immune imprinting remain difficult to model accurately (198). These host-specific factors may influence antibody lineage evolution, immunodominance patterns, and ultimately vaccine-mediated protection.

Computational approaches are increasingly effective at narrowing antigen search space and prioritizing promising candidates, but their current role is better viewed as supporting candidate prioritization and hypothesis generation rather than replacing experimental validation. Until future frameworks more effectively integrate glycan dynamics, conformational thermodynamics, and system-level models of host immunity, empirical *in vitro* and *in vivo* validation will remain essential for linking computational predictions to protective efficacy.

## 7 Conclusion

RSV vaccine research has moved through a series of clear inflection points: early inactivated formulations that failed, the recognition and stabilization of the preF conformation, and, more recently, the incorporation of computational prioritization and iterative optimization frameworks. Resolving the metastable preF trimer and constraining it in its antigenically relevant state has mitigated the historical risk of VAERD, while enabling platforms—including subunit, mRNA, and nanoparticle vaccines—that elicit strong neutralizing responses and measurable protection in both infants and older adults (39, 177).

Even so, stabilizing a single antigenic state does not fully address the problems that remain. Antigenic drift, declining immunity over time, and marked differences between age groups continue to shape vaccine performance. Immune responses in infants tend to be Th2-skewed and developmentally constrained, whereas in older adults they are shaped by immunosenescence and chronic inflammation (199, 200). Such age-dependent immunological differences highlight the need for approaches that extend beyond structural preservation alone, including strategies capable of modulating the quality, durability, and breadth of the immune response.

Recent computational work provides tools for exploring this possibility. PLMs, AlphaFold-informed structural sampling, and learned fitness landscapes are increasingly used together to explore sequence space in a more directed way (26, 27). Computational approaches increasingly permit candidate antigens to be evaluated before experimental testing, although definitive assessment still requires empirical validation. However, current computational models still incompletely capture glycan-mediated shielding, transient conformational ensembles, and the structural complexity of heavily glycosylated viral antigens, underscoring the continued importance of experimental structural and immunological validation (201, 202). At the same time, delivery strategies can be adjusted to favor more appropriate immune polarization across age groups.

The lessons from RSV F extend beyond a single virus. Similar design logic—maintaining preF conformations, preserving vulnerable epitopes, and anticipating escape—has already informed work on influenza hemagglutinin stem immunogens and coronavirus spike proteins (110, 184). With closer coupling between computation and experiment, computational frameworks are increasingly serving to prioritize candidates and accelerate iterative refinement rather than replace empirical validation. The result is not a finished solution, but a more scalable and hypothesis-guided framework for developing vaccine candidates for empirical evaluation. As computational and experimental vaccinology become more tightly integrated, whether computational approaches can meaningfully improve vaccine efficacy remains an open question that will require rigorous prospective validation.
